# Serum amyloid A-mediated neuro-inflammation induces hippocampal neuron apoptosis and postoperative cognitive dysfunction in mice

**DOI:** 10.3389/fphar.2025.1661881

**Published:** 2025-10-28

**Authors:** Xiaochen Fu, Zhenbin Cai, Yuxuan Wu, Yiwen Xu, Ze Gao, Minghui Tan, Jing Wang

**Affiliations:** Department of Orthopedics, The First Affiliated Hospital of Jinan University, Guangzhou, China

**Keywords:** POCD, neuronal inflammation, SAA, NLRP3, apoptosis

## Abstract

**Objective:**

This study aimed to explore whether serum amyloid A (SAA) triggers an inflammatory response by activating the NOD-like receptor protein 3 (NLRP3) pathway, resulting in hippocampal neuron apoptosis and cognitive impairments in mice.

**Methods:**

SAA was applied to BV-2 and HT22 cells to determine the optimal concentration and duration for stimulation. Inflammation-related and differentially expressed genes were identified through mRNA transcriptome sequencing (RNA-seq). Furthermore, apoptosis in hippocampal neurons was detected following treatment with the SAA-pretreated BV-2 cell culture medium. Furthermore, a postoperative cognitive dysfunction (POCD) mouse model was established using internal fixation of tibial fractures, followed by intraperitoneal injection of SAA and MCC950 (a selective NLRP3 inhibitor). Behavioral tests were then conducted to evaluate cognitive dysfunction in mice.

**Results:**

mRNA transcriptome sequencing revealed that SAA led to the upregulation of inflammatory factors, including interleukin-1β (IL-1β). In cultured BV-2 cells, SAA treatment activated the NLRP3 signaling pathway. Additionally, the supernatants from SAA-treated BV-2 cells significantly increased the apoptotic rate in HT22 cells and primary hippocampal neurons. Pharmacological inhibition of NLRP3 using MCC950 reduced this apoptosis. The tibial fracture intramedullary nailing technique effectively established a mouse model of POCD, with SAA administration increasing inflammatory factor levels in the hippocampus of POCD mice and impairing their cognitive abilities. However, treatment with MCC950 significantly alleviated the cognitive dysfunction induced by SAA in the POCD mice.

**Conclusion:**

SAA treatment triggers an inflammatory response through the activation of NLRP3, which subsequently causes hippocampal neuron apoptosis and impairs cognitive function in POCD mice. This dysfunction can be reversed by inhibiting NLRP3 with the administration of MCC950.

## Introduction

Over 60 years ago, the first clinical case report was published, describing an elderly individual who experienced cognitive decline following anesthetic surgery (Bedford, 1955). Clinical manifestations included disorientation, memory loss or inattention. Such symptoms are now referred to as postoperative cognitive dysfunction (POCD) and negatively impact the quality of life of millions of people each year (Wang et al., 2024). Early studies focused on cardiac surgery, possibly because of the potential for hypoxic or ischaemic episodes during this type of surgery (Rasmussen, 2006). It is now believed that POCD can also occur in non-cardiac surgeries, including major thoracic, abdominal, and orthopedic procedures such as hip and knee arthroplasty (Shoair et al., 2015). As POCD seriously affects postoperative treatment and leads to a decrease in quality of life, how to slow down the progression of POCD in elderly patients has become a high concern for clinical practitioners (Varpaei et al., 2024). Cognitive decline is an inevitable aspect of the normal aging process (Murman, 2015). Strong evidence suggests that advanced age is the primary risk factor for the development of POCD, with a previous history of perioperative cognitive impairment or stroke serving as additional risk factors (Netto et al., 2018). Thus, despite a large number of studies in this area, the pathogenesis of POCD is still in the exploratory stage and is not yet conclusive.

Serum amyloid A (SAA) is an acute-phase protein encoded and synthesized by multiple genes in response to temporal changes (Sack, 2018). Under normal conditions, SAA protein is undetectable in the brain; however, SAA gene expression has been observed in the brain tissue of both Alzheimer’s disease (AD) and multiple sclerosis (MS) patients (Liang et al., 1997). In mouse models of traumatic brain injury (TBI), SAA levels can be elevated in both blood and liver early on, and serum SAA may serve as a new, neuroinflammation-based biomarker for acute TBI, with its levels correlating to the severity of the injury (Wicker et al., 2019). It is known that in human macrophages, SAA activates pro-IL-1β synthesis by stimulating Toll-like receptor-2 (TLR2) and Toll-like receptor-4 (TLR4) (Facci et al., 2018). Interestingly, SAA may play a role in transmitting both signals necessary for the production of mature IL-1β. IL-1β is mediated by the NLRP3 inflammasome, histone B, and cysteine-containing aspartate-specific protease caspase-1, and it may contribute to the pathogenesis of neurological diseases (Song et al., 2017). Inhibition or knockout of NLRP3 in microglia prevents the elevation of IL-1β levels (Liu et al., 2022). The NLRP3 inflammatory pathway is a key component of a broader pro-inflammatory pathway, and its regulatory role is still under investigation (Gupta et al., 2024). In addition to this, SAA induces the release of various cytokines from many cell types including monocyte-macrophages, neutrophils and lymphocytes (Sack, 2018). SAA-deficient mice exhibit reduced inflammation and smaller infarct volume during stroke (Yu et al., 2019). These studies suggest that brain injury can trigger a systemic inflammatory response mediated through SAA.

Neuroinflammation is now considered to play a key role in the development of POCD (Peng et al., 2023). Surgical procedures can induce systemic stress responses, leading to the release of neuroendocrine hormones and inflammatory mediators, which disrupt the blood-brain barrier (BBB), activate microglia, and trigger neuroinflammation (Hu et al., 2024). Animal studies suggest that POCD may be caused by BBB dysfunction and neuroinflammation after surgery (He et al., 2012). Activated microglia produce pathological cytokines such as CD11b, IL-1β and brain-derived neurotrophic factor, resulting in a vicious cycle of neuroinflammation (Qiu et al., 2016). The pathological basis of various neurodegenerative diseases lies in the gradual loss of neuronal structure and function, as well as disruption of glial cell homeostasis, leading to cognitive disorders such as dementia (Kampmann, 2024).

Recent years have seen SAA widely used in the diagnosis and prevention of infectious diseases, yet the relationship between SAA, neuroinflammation and POCD remains poorly understood. Here, we administered SAA to BV-2 cells and applied the culture medium to treat hippocampal neurons to assess its impact on neuronal apoptosis. Additionally, we treated POCD model mice with SAA, observed changes in various behavioral indices, and explored the underlying mechanisms.

## Materials and methods

### Cell culture

BV-2 cell line was applied to study microglia in our *in vitro* studies. HT22 cell line was applied to study neurons in our *in vitro* studies. The BV-2 microglial cell line and HT22 cells were both purchased from Procell Life Science& Technology Co.,Ltd (CL-0493, CL-0697, Wuhan, China), which were plated in cell culture plates (Corning Costar, 3516, Cambridge, MA, USA) and cultured with DMEM (Gibco, C119955000BT, Billings, MT, USA) containing 10% fetal bovine serum (Gibco, 10,099, Billings, MT, USA) and maintained in a humidified incubator (Thermo, USA) at 37 C with 5% CO2. We used an NLRP3 inhibitor (MCC950, Medchemexpress) in our experiments. Recombinant human Apo-SAA was purchased from PeproTech (300–13). Lipopolysaccharide (LPS) from *Escherichia coli* O111:B4 was purchased from Sigma-Aldrich.

### Ethics and establishment of POCD model

A total of 40 male C57BL/6 mice (18 months old, weighing 30–35 g) were used in all studies, which were purchased from Laboratory Animal Center of Southern Medical University (Guangzhou, China), were kept in captivity in a room with humidity maintained at 45% and temperature controlled at 22 C, with a light-dark cycle of 12:12 h and free access to laboratory food and water. Mice were kept in the laboratory for 1 week before the start of the experiment to adapt to the environment. The experimental procedures were performed in accordance with the *Requirements for the Care of Laboratory Animals and Guidelines for Their Use* of the First Affiliated Hospital of Jinan University. The study was reviewed by the Animal Ethics Committee of Jinan University on the principle of minimizing pain and suffering of experimental animals. The ethical review number is IACUC-20211029–07. Notably, we’ve made efforts to minimize the number of animals and their suffering.

After the mice were acclimatized for 1 week, we used intraperitoneal anesthesia and tibial fracture surgery as a POCD model. The anesthetic was 1.25% avertin, and the surgery included intramedullary fixation and tibial fracture. After the right lower limb was prepared and sterilized, a longitudinal incision of 0.5 cm was made under the right knee joint to expose the right tibia, and a 1 mL syringe needle of 0.45 mm diameter was inserted into the tibia to expand the medulla. The highest point of the anterolateral curved forward projection of the tibia was found in the upper middle third of the tibia, and the muscle and fascia were separated. After using ophthalmic scissors to dissect the tibial diaphysis, the tibial surface was rinsed with 0.9% saline. The intramedullary fixation pins were cut to length and shaped, then inserted fully into the intramedullary fixation pins and aligned with the fracture line. The incision was then closed layer by layer with 5–0 vicryl thread, and the Sham group of mice received exactly the same anesthesia, incision and closure, and analgesia as the POCD modelling group, but without surgical intervention. At the end of the operation, tibiofibular orthopantomographs were taken, and the resuscitated mice were returned to the animal room.

### Primary hippocampal neuron extraction and culture

Primary hippocampal neurons were cultured as previously described (Tan et al., 2015). The procedure for primary hippocampal neuron culture includes coverslip coating followed by neuron isolation and plating. Hippocampi of postnatal day 1 C57BL/6 mice pups were dissected and incubated with papain (P4762, Sigma–Aldrich, USA) and DNase I (DN25, Sigma–Aldrich, USA) at 37 C for 20 min. The hippocampi were washed with Dulbecco’s Modified Eagle Medium/F12 containing 10% fetal bovine serum (Gibco). The cells were plated on glass coverslips that were previously coated with poly-D-lysine (Sigma–Aldrich, USA) at a density of 1 × 10^4^ cells/cm^2^. Poly-D-Lysine (PDL) can be recycled and used many times. Plates were incubated at 37 C in a humidified 5% CO2 atmosphere overnight, and the initial culture medium was replaced after the cells were attached, with Neurobasal medium (Gibco, Cat No. 21103–049) supplemented with 2% B27 (Gibco, Cat No. 17504–044). To suppress glial cell proliferation, cultures were treated with a low concentration of the mitotic inhibitor cytosine β-D-arabinofuranoside (Ara-C) for 24–48 h between days 2 and 4 post-plating (DIV 2–4). As shown in Figure 5D, BV-2 cells were stimulated with SAA for 24 h. The cell culture medium was then centrifuged, and the supernatant was collected and transferred to hippocampal neurons cultured for 9 days for conditioned medium incubation over 24 h. Glutamate was used as a positive control. The experiment was performed with three independent biological replicates.

### RNA-seq and enrichment analysis

After extracting RNA from each group of samples, cDNA libraries were constructed. Adaptor-ligated cDNA was used for PCR amplification. Products of PCR were purified with AMPure XP system. Library quality was assessed on an Agilent Bioanalyzer 4,150 system. Ultimately, the sequence of the library preparations was examined on an Illumina NovaSeq and 150 bp paired-end reads were generated. The data generated from Illumina NovaSeq platform were used for bioinformatics analysis. All of the analysis was performed using an in-house pipeline from Shanghai Applied Protein Technology. Clean reads were separately aligned to reference genome with orientation mode using HISAT two software to obtain mapped reads (Kim et al., 2015). Feature counts (http://subread.sourceforgenet) is used to work out the number of reads mapped to each gene. The FPKM for each gene is then calculated based on the gene length and the number of reads mapped to that gene. We performed differential DESeq2 expression analysis using the (http://bioconductor.org/packages/release/bioc/html/DESeg2.html), DEGs with |log2FC| > 1 and Padi <0.05 were considered to be significantly different expressed genes (Love et al., 2014). Gene Ontology (GO) and Kyoto Encyclopedia of Genes and Genomes (KEGG) enrichment analysis aimed at differential genes by cluster Profiler R software package can explain their characteristic function and clarify the differences between samples at the gene function level. When the p-value is less than 0.05, it can be concluded that the GO or KEGG function is significantly enriched (Kanehisa et al., 2008).

### Cell counting Kit-8 assay for cell viability

Cell viability was assessed using the CCK8 assay (A CCK-8 kit; Dalian Meilun Biotechnology Co., Ltd) according to the manufacturer’s instructions. BV-2 microglial cells or HT22 cells were seeded in 96-well plates and treated with various concentrations of SAA or co-cultured medium of BV-2 treated with SAA in advance. The cells were then treated with 10% CCK8 and incubated for 1 h at 37 °C in a 5% CO2 incubator. Absorbance was measured at 450 nm.

### Western blotting analysis of cells and tissues

For Western blotting (WB) analysis, cells were washed with cold PBS, scratched of the wells with cell lysis buffer (MB9900, meilunbio, Dalian Meilun Biotechnology Co., Ltd) and protease inhibitors (Beyotime, China). After 30 min of lysis, the solution was centrifuged for 20 min at 12,000 rpm at 4 C and the supernatant was separated by SDS-PAGE and transferred to 0.22 μm polyvinylidene difluoride membranes (PVDF-ISEQ00010, Millipore, USA). The membrane was blocked in 5% BSA (vetec, USA) dissolved with Tris-buffered saline containing 0.1% Tween-20 (TBST) for 1 h. The following primary antibodies were used: anti-NLRP3 (#15101, 1:1000 for WB, Cell Signaling Technology), anti-Caspase-1 (#24232, 1:1000 for WB, Cell Signaling Technology), anti-IL-1β (#12242, 1:1000 for WB, Cell Signaling Technology), anti-Bax (#2772, 1:1000 for WB, Cell Signaling Technology), anti-Caspase-3 (#9662, 1:1000 for WB, Cell Signaling Technology), anti-Bcl-2 (#Ab182858, 1:1000 for WB, Abcam), anti-tubulin (#Ab7291, 1:1000 for WB, Abcam), anti-Glyceraldehyde-3-phosphate dehydrogenase (GAPDH, #5174, 1:1000 for WB, Cell Signaling Technology). Then the membrane was incubated overnight at 4 °C with specific antibody against NLRP3, IL-1β, Caspase-1, Caspase-3, Bax, Bcl-2, Iba-1, Tubulin or GAPDH, respectively. The membranes were then incubated with an HRP-conjugated secondary antibody. The volumes of protein bands were measured, and the values of protein bands were normalized by these of GAPDH or tubulin from the same samples. Data were analyzed in a semi-automated manner using ImageJ software, and Results were confirmed by an independent researcher who did not participate in the current experiments.

### Quantitative real-time polymerase chain reaction (RT-qPCR)

RNAeasy™ animal RNA extraction kit (Beyotime, China) was applied to total RNA extracts of BV-2 cells according to the manufacturer’s instructions. A HiScript^®^ II 1st strand cDNA synthesis kit (+gDNA wiper) (Vazyme, China) was used to reverse transcribe the mRNA for cDNA synthesis. The qPCR was performed using AceQ ^®^ qPCR SYBR green master mix (Without ROX) (Vazyme, China). mRNA expression levels were normalized to those of GAPDH ing a delta-delta-Ct method (Livak and Schmittgen, 2001). All RT-qPCR reactions were repeated at least three times. RNA concentration was measured with the NanoDrop Onec Spectrophotometer (Thermo Scientific, USA) and reverse transcribed into cDNA using the HiScript II qRT Supermix (Vazyme Biotech; R222-01). Gene expression levels of GAPDH, IL-1β and NLRP3 were measured using real-time quantitative PCR on a Quant Studio 5 (Applied Biosystems Thermo Scientific), using the AceQ qPCR SYBR Green Master Mix (Vazyme Biotech; Q131-02). Gene expression levels were normalized to GAPDH. Primers were designed (Table 1) using the Basic Local Alignment Search Tool (BLAST) from the National Center for Biotechnology Information (NCBI).

**TABLE 1:** The primers used.

Gene	Primer	Resrriction
*GAPDH*	Forward	AGA​AGG​TGG​TGA​AGC​AGG​CAT​C
	Reverse	CGA​AGG​TGG​AAG​AGT​GGG​AGT​TG
*IL-1*β	Forward	GTC​ACA​AGA​AAC​CAT​GGC​ACA​T
	Reverse	GCCCATCAGAGGCAAGGA
*NLRP3*	Forward	TGC​TCT​TCA​CTG​CTA​TCA​AGC​CCT
	Reverse	ACA​AGC​CTT​TGC​TCC​AGA​CCC​TAT

### Enzyme-linked immunosorbent assay (ELISA)

BV-2 microglia culture medium was collected and centrifuged (14,000 g, 5 min) to obtain supernatant. Levels of the inflammatory cytokine IL-1β in tissue homogenates or cell supernatants were determined using an ELISA kit (Cusabio, China) according to the manufacturer’s instructions.

### Flow cytometry (annexin V-FITC/PI staining)

BV-2 cells were inoculated into six-well plates, and when the cell density went up to 80%, different concentrations of SAA were added and cultured for 24 h. The medium of BV-2 cells in each well was collected separately. HT22 cells were inoculated into six-well plates, and make sure the cells were observed to be in good condition. Medium collected from BV-2 cells was transferred to each well in turn, and the incubation was continued for 24 h. Then the HT22 cells were digested with trypsin, and the digestion was terminated by centrifugation of fresh medium with serum, and the cells were resuspended with PBS and collected in ep tubes. The cells were centrifuged at 2000 rpm for 5 min and the supernatant was discarded. Add 100 μL of Binding Buffer to the ep tube and mix well. Add 2.5 μL of Annexin V-FITC, then 2.5 μL of PI and keep it out of the light for 15 min. Transfer the sample to flow-through tube and run. Collect data and plot.

### Immunofluorescence assay

Mice were terminally anesthetized and transcardially perfused with ice-cold 0.9% saline followed by 4% paraformaldehyde (PFA). Brains were carefully removed and post-fixed in the same 4% PFA solution for 24 h at 4 C before being cryoprotected in paraffin embedding. Following dehydration through a graded ethanol series, tissues were cleared in xylene to remove alcohol and facilitate paraffin infiltration. Liquid paraffin was used to replace the hyaluronan in it and immersed into the tissue for support. Tissue blocks were embedded well by liquid paraffin and rapidly frozen until solidified into shape and then cut into 4 μm slices. The slices were floated in warm water at 45 C, picked up by clean slides, labelled with the group and date, and placed on a section holder in an oven at 65 C for 2 h. The slices were dewaxed and hydrated in xylene and ethanol at different concentrations, and permeabilized with 0.5% Triton X-100 in PBS to facilitate antibody penetration. Then they are sequentially closed, incubated with antibody (#Ab178847, anti- Iba-1, 1:8000, Abcam), and blocked. After the sections were dried, they were observed under a microscope (Leica DMi8) to obtain fluorescence images.

### Behaviors assessment

All of the behavioral tests were performed in a dark, quiet, and soundproof room with a comfortable temperature. All behavioral measurements were recorded using a tracking system (XinruanTechnology, Shanghai, China). Each behavioral test was performed by two people who were blinded to the groups.

### Novel object recognition (NOR)

After the acclimatization period, a pair of identical objects was placed and fixed in the box, and the exploration times of the left object and right object were recorded for each mouse within 10 min. After the mice were removed from the box, every corner of the box was wiped with alcohol to remove any residual urine and faeces. During the test period, two different objects were placed and fixed in the box. One of them was a novelty object and the other was identical to the one used before. The mice were placed in the box in turn with their backs to the two objects and the distance between the mice and the objects was the same.

The Supermaze program was turned on using the head-body-tail three-point method to identify and record the exploration time and frequency for the familiar object and for the novel object. Every corner of the box was wiped with alcohol for residual urine and faeces before the next mouse was placed in the experiment. Only when the mice sniffed or licked the objects were characterized as exploratory activities. Cognitive function was assessed by the difference in the spending time and the frequency that mice explored the old and new objects, which was statistically analyzed using the cognitive index. Mice with poor cognitive function showed a lack of interest in the new object, whereas mice with normal cognitive function showed a marked interest in the new object, as evidenced by a higher time and number of explorations of the new object compared to the old object.

### Y-maze test

The YMT was used to assess short-term spatial working memory. The apparatus consists of three white acrylic arms (40 cm × 5 cm × 10 cm) separated at an angle of 120°. Each mouse was gently placed into the distal end of one of the random arms, and then the mouse’s access to the arms was recorded over a 10-min period. The total number of times and the order in which each mouse entered each arm were recorded using the Supermaze program with the center-of-gravity recognition method, and the correct alternation response was judged to be correct when three entries were made into arms that were not the same, e.g., BCA, ABC.

The percentage of correct voluntary alternations (%SA) was calculated as follows: %SA = number of correct arm entries/(total number of arm entries - 2) × 100%. Mice with fewer than 15 alternations during the test period are not counted in the final data. The mice were returned to their cages at the end of the test, and before proceeding to the next experiment, the mice were wiped with paper towels to remove any residual urine and faeces, and the arms were sprayed with 75% alcohol to remove the odour from the Y-maze.

### Image analyses and statistical analyses

All the above experimental operations were repeated more than 3 times in each group according to the group, and cell immunofluorescence staining was performed in five visual fields in each group. To test the representativeness of sampling to the population, all data in this experiment were represented by Mean ± SD, GraphPad Prism eight was used as statistical tool, and one-way ANOVA of variance was used as statistical method. *P* < 0.05 was considered to have statistical significance, in which * represented *P* < 0.05 and ** represented *P* < 0.01. *** means *P* < 0.001.

## Results

### Transcriptomic analysis reveals SAA-Induced upregulation of inflammatory and cytoskeleton-related genes

**FIGURE 1:**
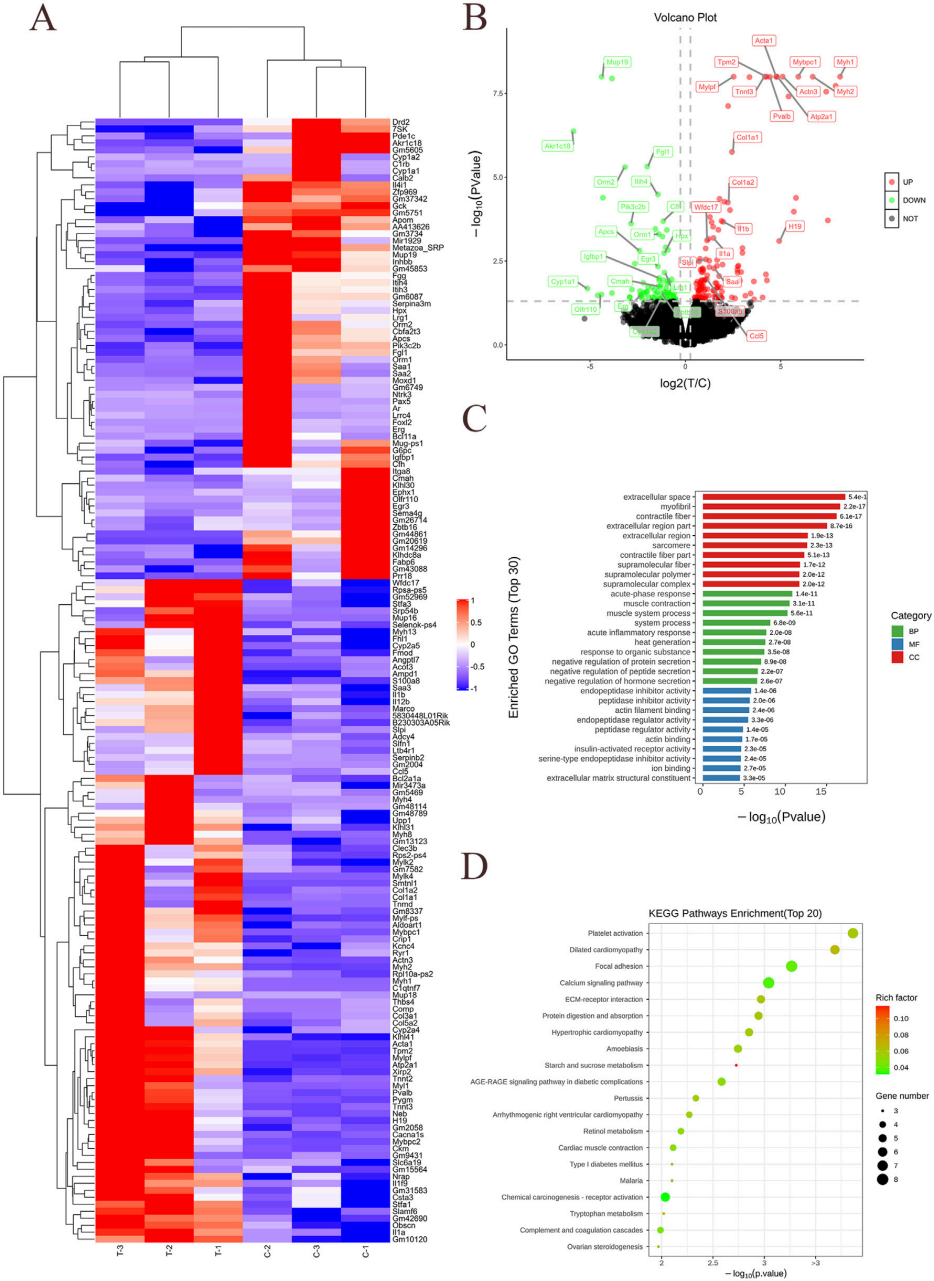
mRNA transcriptomic sequencing and analysis of SAA-stimulated BV-2 cells. **(A)** Heatmap showing hierarchical clustering of differentially expressed genes between control and SAA-treated groups. **(B)** Volcano plot illustrating the distribution of differentially expressed genes (DEGs). **(C)** Gene Ontology (GO) enrichment analysis of DEGs presented as a bar graph. The horizontal axis represents the -log_10_ (Pvalue), and the terms are arranged in ascending order of P value (i.e., from most to least significant). The three major GO categories are indicated with different colored bars: green for BP, blue for MF, red for CC. The vertical axis shows the detailed functional descriptions of the GO terms. **(D)** Kyoto Encyclopedia of Genes and Genomes (KEGG) pathway enrichment analysis of DEGs presented as a bubble chart. Data represent three biological replicates per group (n = 3). The vertical axis represents the names of the pathways. The horizontal axis represents the P-value corresponding to each pathway. The color of the dots represents the size of the Rich factor—darker red indicates a larger value. The size of the dots represents the number of differentially expressed genes contained in each pathway.

We utilized human recombinant Apo-SAA (50 μg/mL, prepared by dissolving 50 μg of lyophilized powder in 1 mL PBS), a consensus protein corresponding to human Apo-SAA1α, which typically circulates in association with HDL (Yu et al., 2019). To elucidate the molecular mechanisms underlying SAA-induced neuroinflammation, RNA sequencing (RNA-seq) was performed to identify differentially expressed genes (DEGs) in BV-2 cells following SAA stimulation. Cells were treated with SAA for 24 h, while PBS-treated cells served as controls. Each group included three biological replicates. Sequencing libraries were constructed and aligned to the reference genome using HISAT2. Transcriptomic analysis revealed substantial gene expression changes in response to SAA stimulation (Figure 1A). Differentially expressed gene (DEG) clustering is used to evaluate the expression patterns of DEGs across different sample groups. Based on the similarity of gene expression levels in each sample, genes are clustered to intuitively visualize their expression profiles across various samples, thereby facilitating the extraction of biologically relevant information. The clustering analysis results are shown in the figure below. Each column represents a sample, and each row represents a gene. Red indicates upregulation, and blue indicates downregulation. The dendrogram at the top represents sample clustering: the closer two sample branches are, the more similar the expression patterns of all DEGs between those samples. The dendrogram on the left represents gene clustering: the closer two gene branches are, the more similar their expression levels. “C” represents the blank control group, and “T” represents the experimental group. A total of 186 mRNAs were differentially expressed (fold change ≥2.0, P < 0.05), with 119 upregulated and 67 downregulated genes compared to the control group (Figure 1B). A volcano plot provides a visual representation of the distribution of differentially expressed genes (DEGs) and their up- or downregulation between two samples or sample groups. As shown in the figure, the x-axis represents the fold change in gene expression between different experimental conditions or samples, while the y-axis indicates the statistical significance of the change in gene expression. Each point in the plot corresponds to an individual gene. Black dots represent genes with no significant difference in expression, red dots denote significantly upregulated genes, and green dots indicate significantly downregulated genes. Notably, several pro-inflammatory cytokines such as IL-1β, as well as genes associated with actin cytoskeletal dynamics, were significantly upregulated.

Gene Ontology (GO) enrichment analysis suggested that SAA may exert its pro-neuroinflammatory effects via biological processes including the acute phase response, muscle contraction, and regulation of endopeptidase inhibitor activity, among others (Figure 1C). As mentioned by the reviewers, the Gene Ontology (GO, http://www.geneontology.org/) is a standardized functional classification system that provides a dynamically updated controlled vocabulary to describe the attributes of genes and gene products in organisms across three aspects: involved Biological Processes (BP), Molecular Functions (MF), and Cellular Components (CC).

In this experiment, the significance enrichment analysis of GO functional annotations for differentially expressed genes was performed using Fisher’s exact test to evaluate the significance level of protein enrichment in specific GO terms. Functional enrichment analysis of differentially expressed genes compares all such genes against the reference genome based on GO annotation results. Through Fisher’s exact test, the significance of differences was assessed to identify significantly enriched functional categories among all differentially expressed proteins (P value <0.05). This analysis, conducted at the level of individual GO terms, directly reveals the overall functional enrichment characteristics of all differentially expressed genes. These significantly enriched GO terms often reflect biological functions of key interest to researchers. The results of the GO functional enrichment analysis for differentially expressed genes in this experiment are presented in the table below (Table 2).

**TABLE 2:** Table of enrichment analysis results.

GO_ID	Term	Category	Test	Ref	P Value	FDR
GO:0030016	Myofibril	CC	10	234	4.47e-14	5.19e-11
GO:0043292	Contractile fiber	CC	10	245	7.08e-14	5.19e-11
GO:0030017	Sarcomere	CC	9	213	1.16e-12	5.64e-10
GO:0044449	Contractile fiber part	CC	9	223	1.75e-12	6.40e-10
GO:0015629	Actin cytoskeleton	CC	11	499	2.53e-12	7.40e-10

Annotation: GO_ID: The unique identifier in the Gene Ontology database. Term: Description of the Gene Ontology function. Category: The class of the GO, term (BP, MF, or CC). Test: Number of differentially expressed genes associated with the term. Ref: Number of background genes associated with the term. Pvalue: Statistical significance level of the enrichment; a term is considered significantly enriched when P value <0.05. FDR: Adjusted P value.

In this experiment, the top 10 most significantly enriched terms from each of the three major GO categories are presented in a bar chart. A total of 30 functional terms are displayed. If any category contains fewer than 10 terms, the remaining slots are supplemented with terms from other categories. If the total number of enriched terms is fewer than 30, all available terms are shown. The result is illustrated in the figure below.

Zhang et al. (Shoair et al., 2015) established a laparotomy model in aged mice and administered SB-3CT—a chemical endopeptidase inhibitor (25 mg kg^-1^, i. p.)—24 h post-surgery. They observed a 58% reduction in hippocampal MMP-9 activity, a 45% decrease in IL-1β levels, and a 31% shortening of escape latency in the Morris water maze. These findings suggest that inhibiting endopeptidase activity may improve Postoperative Cognitive Dysfunction (POCD) by suppressing neuroinflammation.

In endothelial cell-specific laminin-α5 knockout (α5-ECKO) mice, intracerebral hemorrhage or trauma significantly exacerbated blood-brain barrier (BBB) disruption, leading to extensive leakage of peripheral IgG and fibrinogen into the hippocampus, activation of microglia/macrophages, aggravated neuroinflammation, and worsened cognitive function (Varpaei et al., 2024). This study provided the first *in vivo* evidence that endothelial laminin-α5 is a critical extracellular matrix (ECM) component for maintaining cerebrovascular homeostasis and limiting post-hemorrhagic brain injury, offering new therapeutic insights targeting the basement membrane-integrin axis in stroke treatment.

Preoperative insulin resistance serves as a strong predictor for POCD development. Targeted prevention and treatment strategies against insulin resistance may represent effective interventions for patients at risk of POCD. Murman (2015) conducted a prospective observational clinical study including 124 patients aged 60 years or older scheduled for gastrointestinal surgery. Insulin resistance was assessed using the Homeostatic Model Assessment for Insulin Resistance (HOMA-IR). Multivariate logistic regression and receiver operating characteristic (ROC) curve analyses were employed to evaluate the relationship between HOMA-IR and POCD. Fifty-one patients (41.1%) were diagnosed with POCD on postoperative day 7. The preoperative HOMA-IR value was significantly higher in the POCD group than in the non-POCD group. Additionally, CRP and TNF-α levels were significantly elevated at all postoperative time points in the POCD group (P < 0.05). In a related animal study, Netto et al. (2018) demonstrated that daily intranasal administration of insulin (1.75 U/mouse/day) for 3 days prior to anesthesia completely prevented anesthesia-induced deficits in spatial learning and memory, as well as long-term neurobehavioral alterations tested up to 65 days after exposure in 3xTg-AD mice. These results indicate that both aging and Alzheimer’s disease-like pathology increase susceptibility to post-anesthesia cognitive impairment, and that intranasal insulin treatment may protect against anesthesia-induced cognitive decline.

Kyoto Encyclopedia of Genes and Genomes (KEGG) pathway enrichment further implicated platelet activation, dilated and hypertrophic cardiomyopathy, focal adhesion, calcium signaling, extracellular matrix (ECM)-receptor interaction, and protein digestion and absorption as potential pathways involved in the SAA-mediated response (Figure 1D). KEGG is a database that integrates genomic, chemical, and systemic functional information. Its most well-known component is KEGG PATHWAY, which provides manually drawn pathway maps involving cellular processes, biological systems (e.g., immune system, nervous system), disease pathways, and more. These maps help researchers understand the roles of genes in high-level functional pathways.

The method of KEGG pathway enrichment analysis is similar to GO enrichment analysis. It takes KEGG pathways as units and uses the reference genome as a background. Fisher’s exact test is applied to calculate the significance level of gene enrichment in each pathway, thereby identifying significantly affected metabolic and signal transduction pathways. The results are shown in the table below (Table 3).

**TABLE 3:** Table of KEGG enrichment analysis results.

ID	Description	Test	Ref	P Value	FDR
mmu04260	Cardiac muscle contraction	2	87	2.59e-03	2.64e-02
mmu05410	Hypertrophic cardiomyopathy	2	91	2.84e-03	2.64e-02
mmu05412	Arrhythmogenic right ventricular cardiomyopathy	2	77	2.04e-03	2.64e-02
mmu05414	Dilated cardiomyopathy	2	94	3.02e-03	2.64e-02
mmu04261	Adrenergic signaling in cardiomyocytes	2	152	7.73e-03	5.41e-02

Annotation: ID: Unique pathway accession number in the KEGG, database. Description: Descriptive title of the KEGG, pathway. Test: Number of differentially expressed genes (DEGs) annotated to the pathway. Ref: Number of background genes annotated to the pathway. Pvalue: P-value indicating the statistical significance of enrichment. FDR: Adjusted p-value using the Benjamini–Hochberg (BH) method for multiple testing correction.

The scatter plot of KEGG enrichment analysis for differentially expressed genes is a visual representation of the KEGG enrichment results, as shown in the figure below. In this experiment, the top 20 most significantly enriched pathways are displayed in the plot. In this plot, the degree of KEGG enrichment is measured by the Rich factor, FDR (False Discovery Rate), and the number of genes enriched in a given pathway. The Rich factor is defined as the ratio of the number of differentially expressed genes mapped to a specific pathway to the total number of genes annotated to that pathway. The P-value indicates the significance of pathway enrichment, ranging from [0, 1]. A value closer to zero indicates more significant enrichment.

As shown in the figure, these genes are significantly enriched in pathophysiological processes such as platelet activation, focal adhesion, calcium signaling pathway, protein digestion and absorption, as well as starch and sucrose metabolism. They are highly associated with the AGE-RAGE signaling pathway in diabetic complications and type 1 diabetes mellitus, and closely related to hereditary cardiomyopathies such as hypertrophic cardiomyopathy (HCM) and dilated cardiomyopathy (DCM). Existing literature indicates that elderly diabetic patients undergoing hip replacement surgery face a significantly increased risk of postoperative cognitive dysfunction (POCD). In a large cohort of 3,272 patients undergoing total hip arthroplasty, diabetes mellitus was identified as a key risk factor for PND (Perioperative Neurocognitive Disorders), along with advanced age, cerebrovascular disease, and low hemoglobin levels [8]. While no direct evidence currently links the specific terms “DCM” or “HCM” to “POCD”, substantial indirect evidence suggests that both DCM and HCM can elevate the risk of cognitive impairment through mechanisms such as chronic heart failure, hypoperfusion, inflammation, and microemboli [9, 10]. Consequently, when these patients undergo surgery (including non-cardiac procedures), their likelihood of developing POCD may be higher than that of the general population [11].

### SAA activates the NLRP3 signaling pathway in BV-2 cells in a dose- and time-dependent manner

**FIGURE 2:**
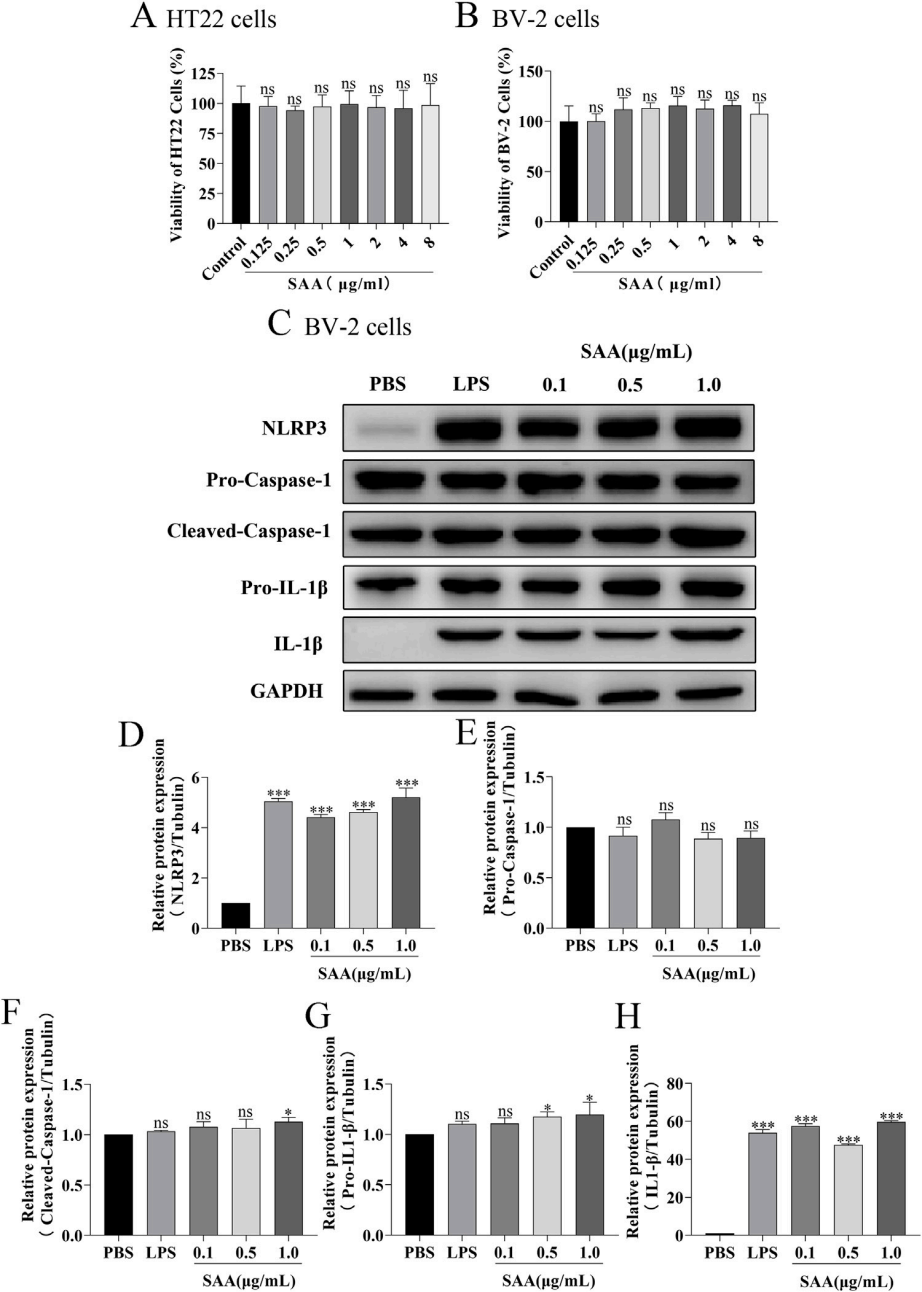
SAA activates the NLRP3 signaling pathway in BV-2 cells in a concentration-dependent manner. **(A,B)** Cytotoxicity analysis of various concentrations of SAA on BV-2 and HT22 cells using the CCK8 assay. **(C)** BV-2 cells were treated with increasing concentrations of SAA for 6 h, followed by Western blot analysis of NLRP3 pathway-related proteins. **(D–H)** Quantitative analysis of protein band intensities corresponding to panel **(C)**. Data are presented as mean ± SD (n ≥ 3). Statistical analysis was performed using one-way ANOVA. *P < 0.05, **P < 0.01, ***P < 0.001.

**FIGURE 3:**
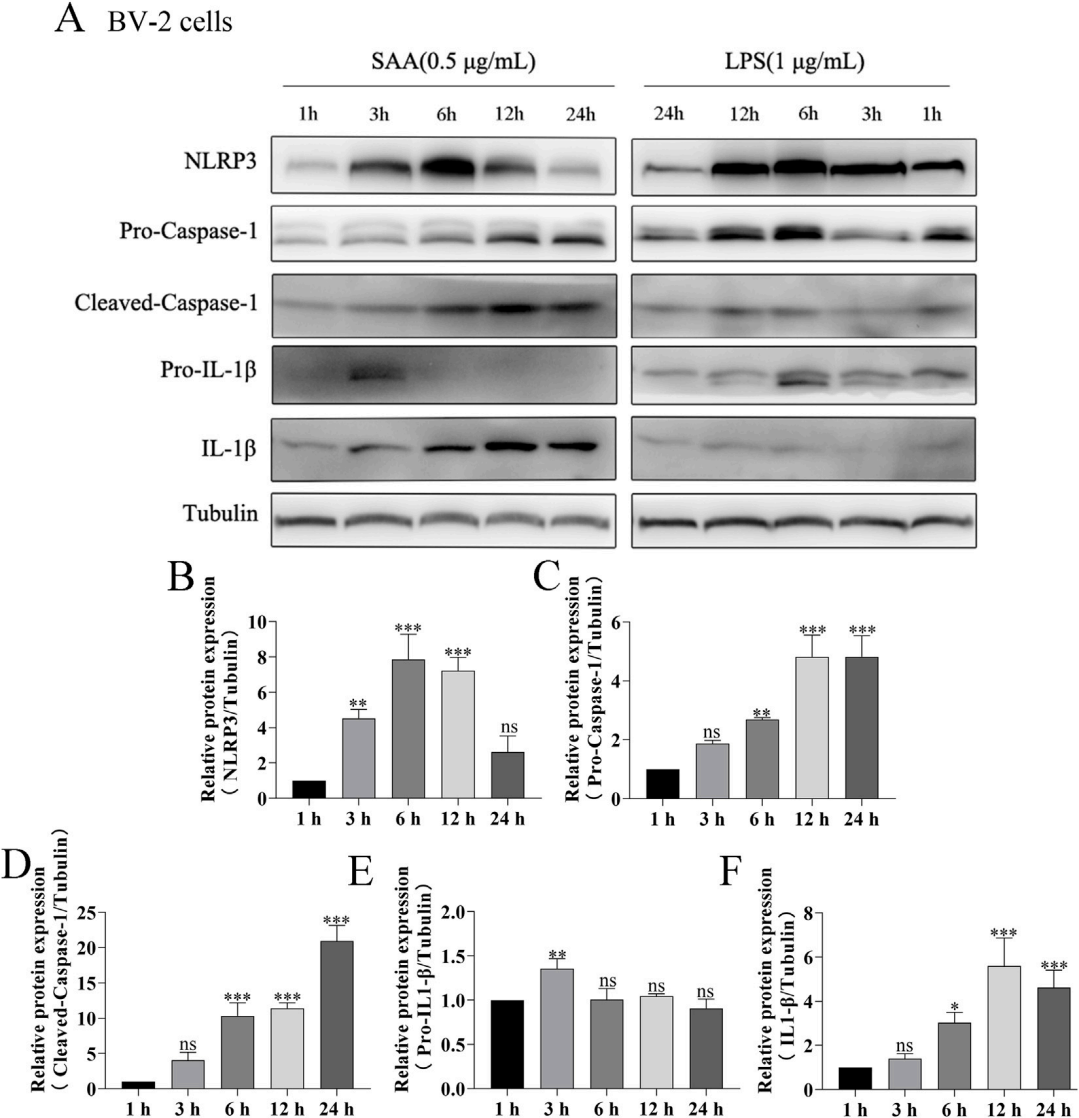
SAA activates the NLRP3 signaling pathway in BV-2 cells in a time-dependent manner. **(A)** BV-2 cells were treated with 0.5 μg/mL SAA for 1, 3, 6, 12, and 24 h. LPS (1 μg/mL) was used as a positive control. Protein lysates were collected for Western blot analysis of NLRP3 pathway-related proteins. **(B–F)** Quantitative analysis of protein band intensities from panel **(A)**. Data are presented as mean ± SD (n ≥ 3). Statistical significance was determined using one-way ANOVA. *P < 0.05, **P < 0.01, ***P < 0.001.

**FIGURE 4:**
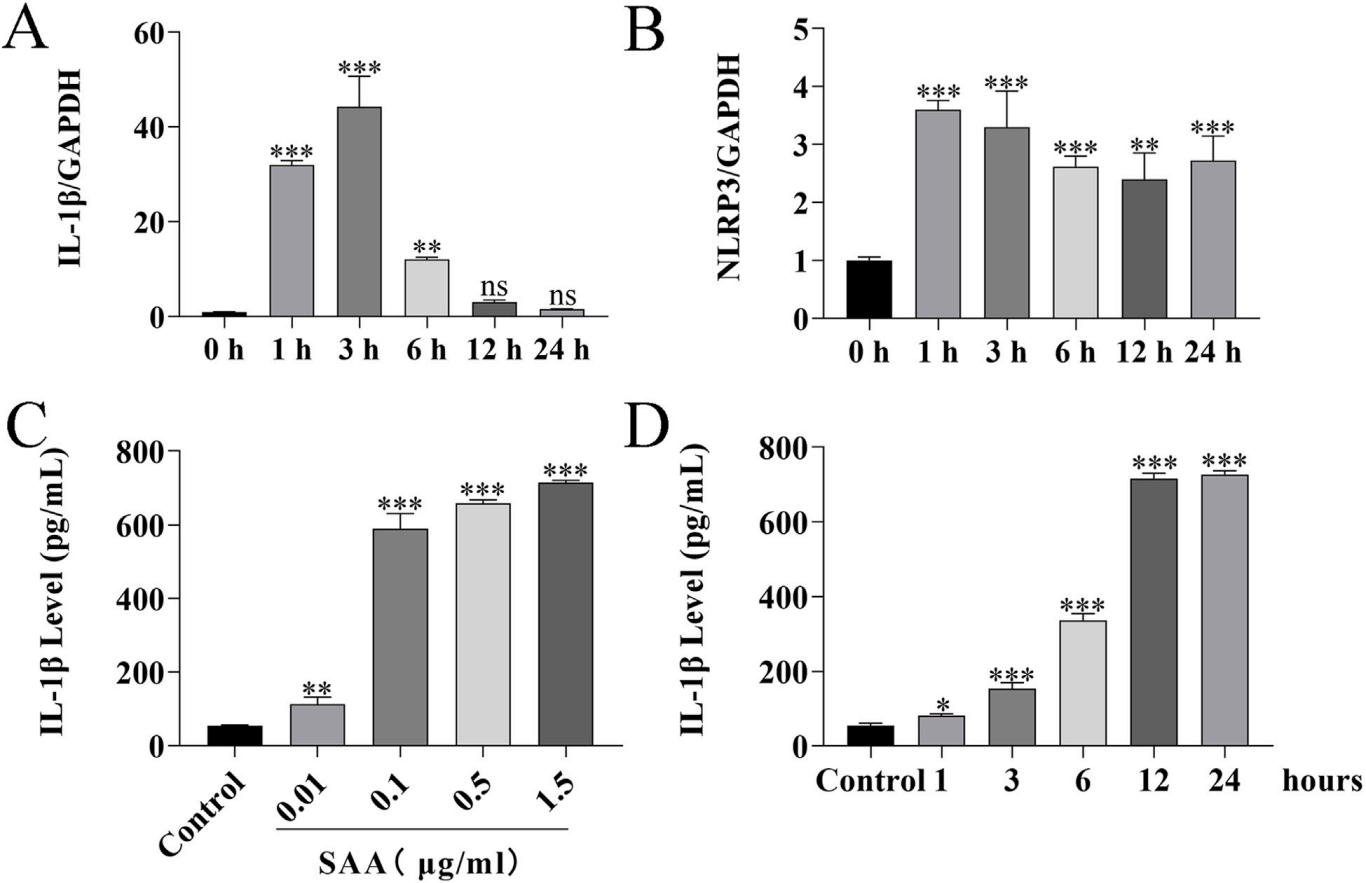
SAA treatment induces the production of inflammatory factors in BV-2 cells. **(A,B)** BV-2 cells were treated with 0.5 μg/mL SAA for 1, 3, 6, 12, and 24 h. Total RNA was extracted at each time point and analyzed by RT-qPCR to assess the expression of inflammation-related genes. **(C,D)** Culture supernatants from BV-2 cells treated with 0.5 μg/mL SAA for the indicated time points (1, 3, 6, 12, and 24 h) were collected and analyzed for IL-1β levels using ELISA. Data are presented as mean ± SD (n ≥ 3). Statistical analysis was performed using one-way ANOVA. *P < 0.05, **P < 0.01, ***P < 0.001.

Here, lipopolysaccharide (LPS) was used as a positive control, while PBS served as a vehicle control. Cytotoxicity of SAA was assessed in BV-2 and HT22 cells using the CCK8 assay. Results demonstrated that SAA concentrations ranging from 0.125 to 8 μg/mL did not exhibit significant cytotoxic effects in either cell line (Figures 2A,B). Subsequently, BV-2 cells were exposed to SAA at various concentrations (0.1, 0.5, 1.0 μg/mL) and for different durations (1 h, 3 h, 6 h, 12 h, and 24 h). PBS- and LPS-treated groups were included as negative and positive controls, respectively. Western blot analysis revealed that SAA activated the NLRP3 signaling pathway in both a dose- and time-dependent manner (Figures 2C–H, 3A–F). Notably, at 1.0 μg/mL, SAA elicited a comparable level of NLRP3 activation to that of LPS. RT-qPCR was employed to assess the mRNA levels of NLRP3 pathway-associated genes following SAA stimulation. Peak expression levels of NLRP3 and IL-1β mRNA were observed at 1 h and 3 h post-treatment, respectively (Figures 4A,B). Given that IL-1β is a secreted cytokine, ELISA was used to quantify its levels in the culture supernatant. Results showed that SAA induced IL-1β secretion in a dose- and time-dependent manner (Figures 4C,D). Collectively, these findings indicate that SAA significantly promotes the expression and activation of the NLRP3 inflammasome and enhances IL-1β transcription and secretion.

### Conditioned medium from SAA-Stimulated BV-2 cells induces apoptosis in HT22 cells and primary hippocampal neurons

**FIGURE 5:**
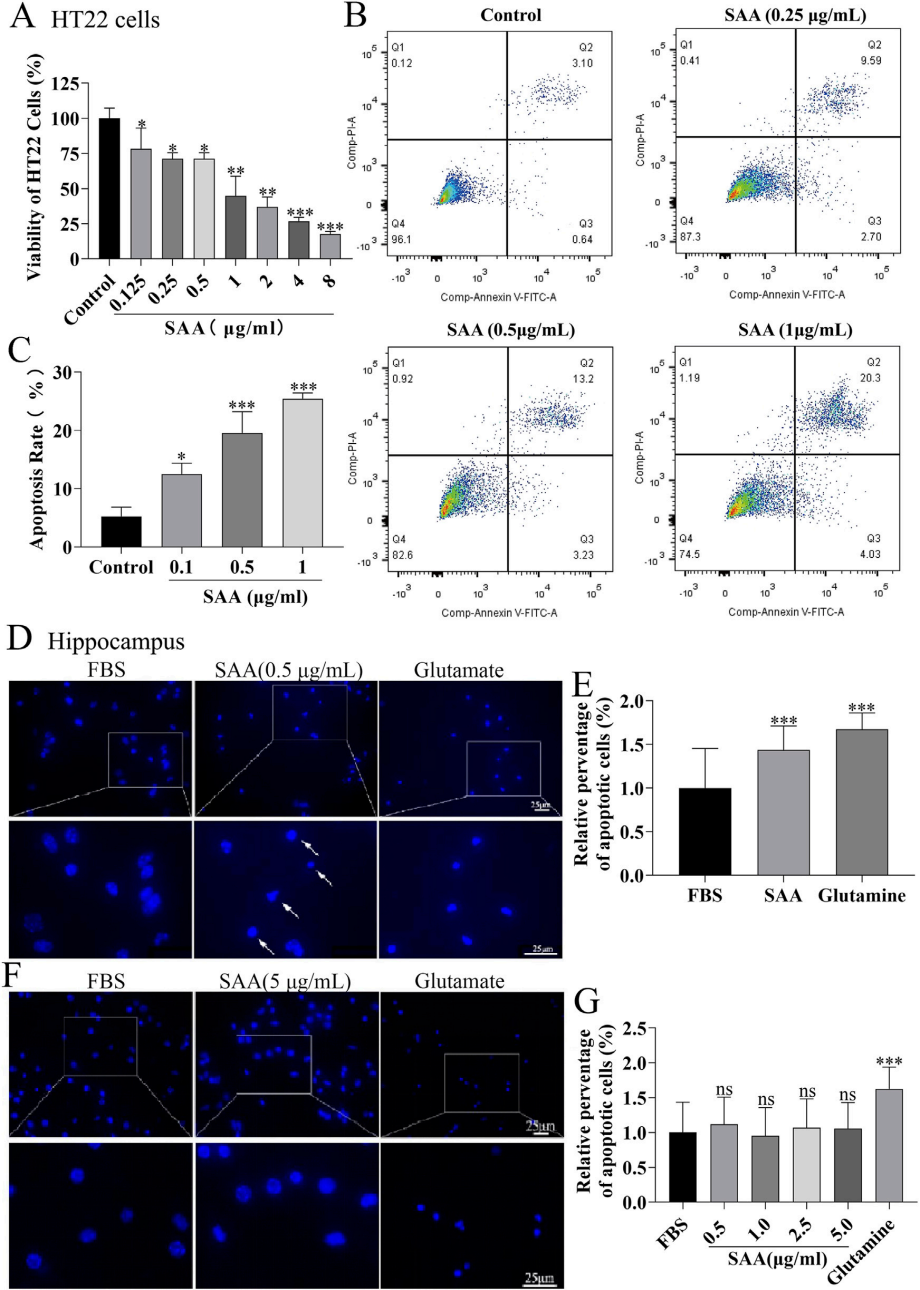
SAA-stimulated BV-2 microglia-derived inflammatory mediators promote HT22 cell apoptosis and neuronal apoptosis​​. ​**​(A)**​​ HT22 cells were treated with conditioned medium (CM) from BV-2 cells stimulated with increasing concentrations of SAA (0, 0.1, 0.5, 1.0 μg/mL) for 24 h, and cell viability was assessed. ​​**(B)**​​ Apoptosis of HT22 cells induced by CM from SAA-pre-stimulated BV-2 cells (0.5 μg/mL, 24 h) was quantified by flow cytometry. ​​**(C)**​​ Statistical analysis of HT22 apoptosis rates from panel **(B)** ​​**(D)**​​ Primary hippocampal neurons were cultured in CM from BV-2 cells pre-treated with 0.5 μg/mL SAA for 24 h, followed by immunostaining with anti-NEU antibody (neuronal marker) and Hoechst 33,342 (nuclear staining). ​​**(E)**​​ Primary hippocampal neurons treated directly with 0.5 μg/mL SAA for 24 h were similarly immunostained as a control. ​​**(F,G)**​​ Neuronal apoptosis was quantified by measuring nuclear condensation (Hoechst-positive area) and expressed as relative apoptosis rate. Data are presented as mean ± SD (n ≥ 3 for A–C; n ≥ 110 neurons/group for **(D–G)**. All data were expressed as Mean ± SD, one-way-ANOVA, *P < 0.05, **P < 0.01, ***P < 0.001, n = 3/group.

**FIGURE 6:**
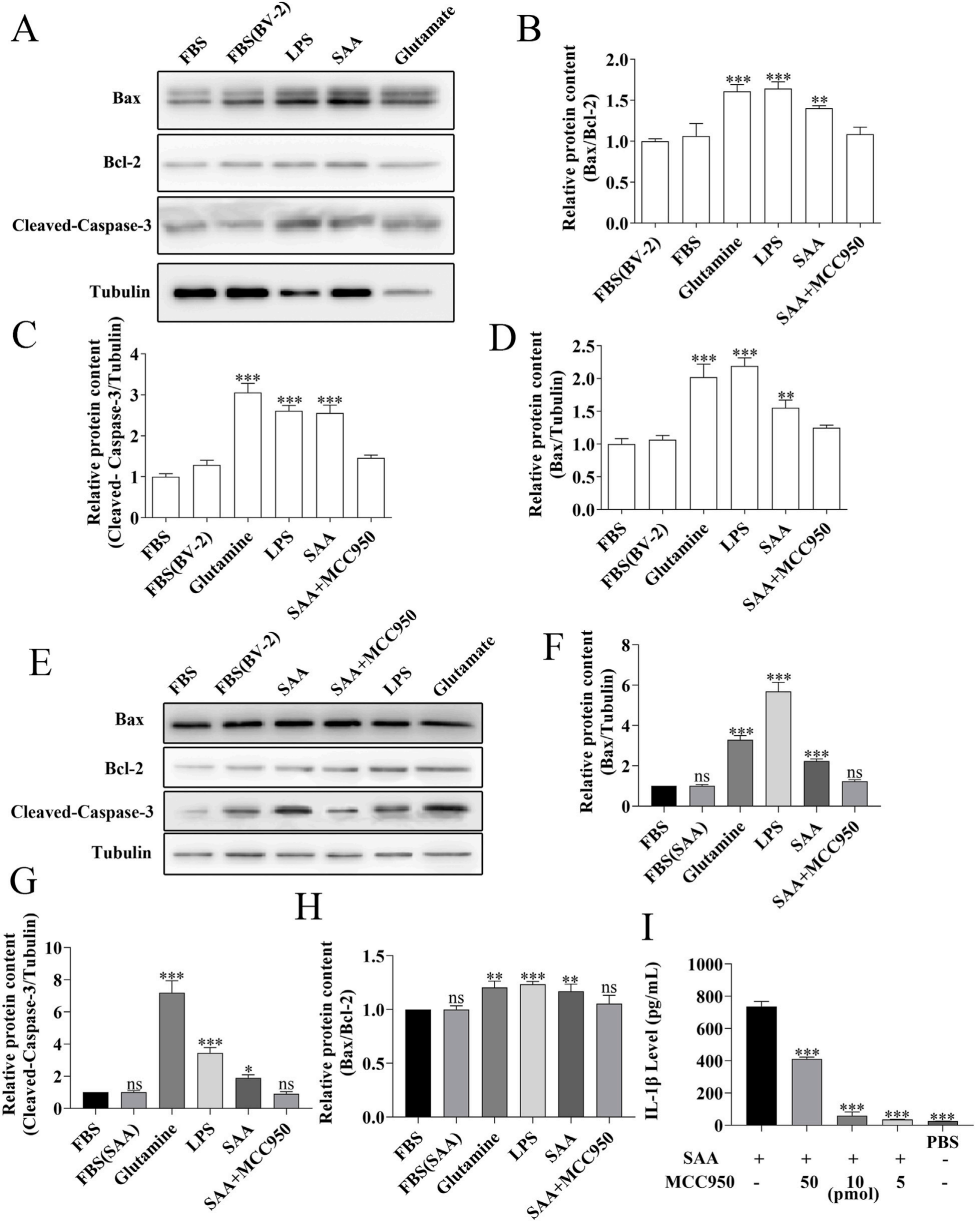
MCC950 inhibits SAA-activated microglia-mediated neuronal apoptosis​​. **​​(A,E)**​​ Primary hippocampal neurons (9 DIV) were treated for 24 h with conditioned medium from BV-2 cells pre-stimulated with 0.5 μg/mL SAA. Apoptosis-related proteins were analyzed by Western blotting. ​​**(E)**​​ shows representative blots. ​​**(B–D,F–H)**​​ Quantitative analysis of: ​​**(B)**​​ cleaved-caspase-3, ​​**(C)**​​ Bax, and ​​**(D)**​​ Bcl-2 expression levels, with ​​**(F–H)**​​ showing Bax/Bcl-2 ratio. Gray values were measured using ImageJ and normalized to β-actin. ​​**(I)**​​ BV-2 cells were treated for 24 h with (i) 0.5 μg/mL SAA, (ii) SAA + MCC950 (50 pmol/L), (iii) SAA + MCC950 (10 pmol/L), (iv) SAA + MCC950 (5 pmol/L), or (v) PBS control. IL-1β levels in conditioned media were measured by ELISA. All data were expressed as Mean ± SD, one-way-ANOVA, *P < 0.05, **P < 0.01, ***P < 0.001, n = 3/group.

To evaluate the neurotoxic potential of inflammatory mediators released by SAA-stimulated microglia, conditioned media (CM) from BV-2 cells treated with increasing concentrations of SAA were applied to HT22 cells. Cell viability assays revealed a concentration-dependent reduction in viability (Figure 5A). To further characterize the type of cellular damage, apoptosis in HT22 cells was assessed using flow cytometry after 24-h exposure to CM from BV-2 cells stimulated with 0.25, 0.5, and 1.0 μg/mL SAA. The apoptotic rate increased with higher SAA concentrations (Figures 5B,C). Additionally, primary mouse hippocampal neurons were examined for nuclear morphological changes. Neurons were stained with a neuronal marker (NEU) and Hoechst 33,342. Microscopy revealed decreased nuclear fluorescence intensity in neurons treated with SAA-conditioned media, suggesting nuclear condensation consistent with apoptotic changes (Figures 5D–G), similar to those observed in glutamate-induced neuronal injury. To determine whether the pro-apoptotic effects were specific to SAA-mediated microglial activation, primary hippocampal neurons were exposed to CM from SAA-stimulated BV-2 cells. Compared to controls and glutamate-treated groups, CM from SAA-stimulated BV-2 cells markedly increased the expression of pro-apoptotic proteins Bax and cleaved Caspase-3 (Figure 6A), as quantified in Figures 6B–D. These findings confirm that the inflammatory microenvironment induced by SAA promotes neuronal apoptosis.

### MCC950 alleviates the apoptosis of primary hippocampal neurons induced by the SAA-treated conditioned medium from BV-2 cells

MCC950 is known to be a potent small molecule inhibitor of NLRP3 inflammasome that inhibits inflammatory signaling (Li et al., 2022). To further demonstrate that SAA mediates hippocampal neuronal apoptosis through the NLRP3 pathway, we included an SAA + MCC950 group in the experiment. The results are presented in Figure 6E, with Figures 6F–H showing the quantitative statistical data. The data revealed MCC950 significantly mitigated the pro-apoptotic effects of SAA-pre-stimulated BV-2 cell supernatant on hippocampal neurons. We measured the concentration of IL-1β in the supernatant using ELISA after 24 h of treatment. The results showed that MCC950 significantly reduced IL-1β release (Figure 6I). These data suggest that pharmacologically inhibition of NLRP3 pathway alleviates the apoptosis of primary hippocampal neurons by SAA-treated conditioned inflammatory medium.

### Tibial fracture with intramedullary pinning induces hippocampal inflammation and establishes a reliable POCD model in aged mice

**FIGURE 7:**
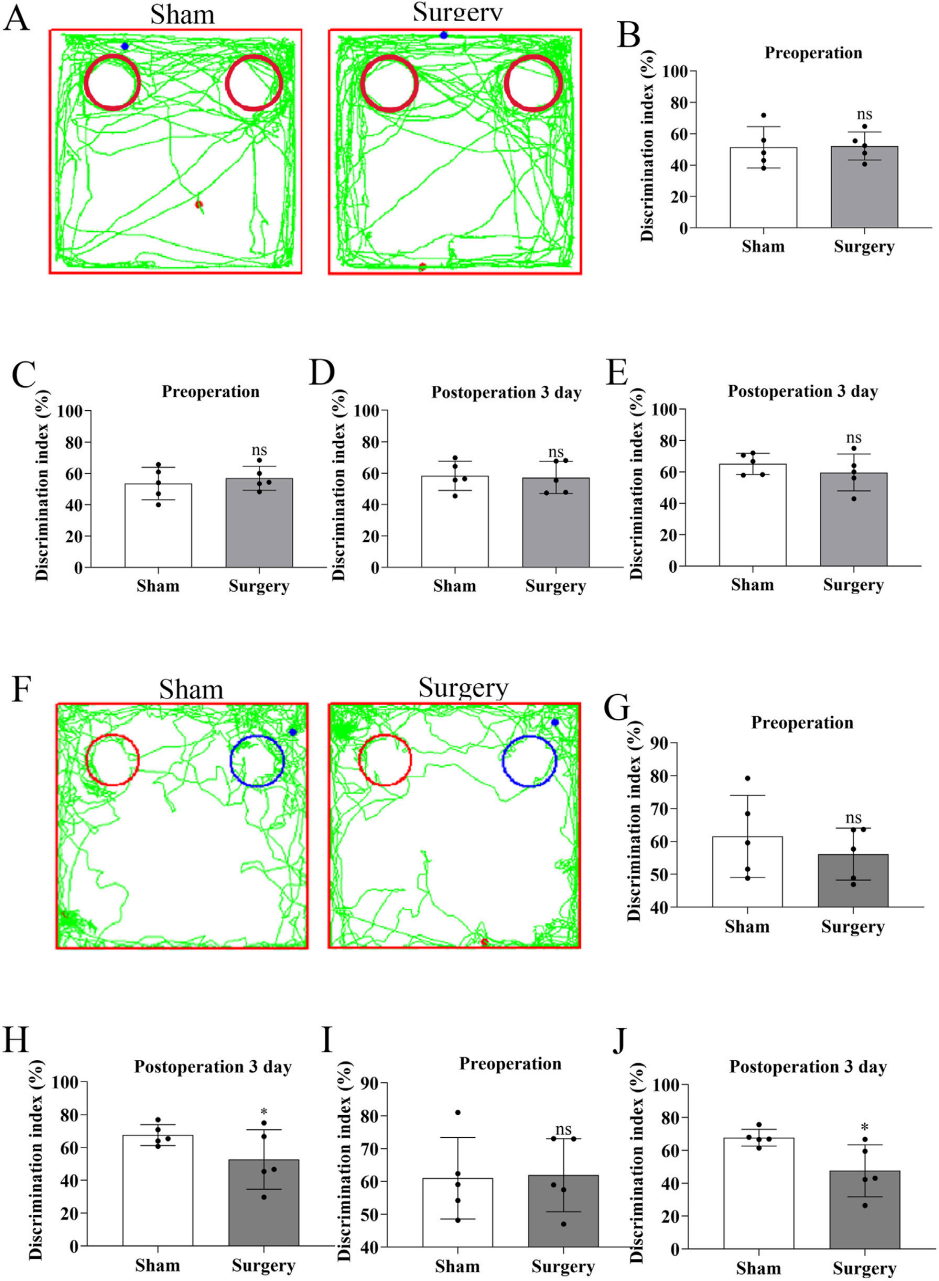
Cognitive assessment of postoperative cognitive dysfunction (POCD) in aged mice using novel object recognition test​​. ​​**(A)**​​ Representative movement trajectories showing head probe interactions with identical objects (red circles) at postoperative day 3 (POD3) in both experimental groups. ​​**(B,D)**​​ Comparison of object exploration time between identical objects preoperatively (day −1) versus POD3. ​​**(B)**​​ shows absolute time differences, while ​​**(D)**​​ presents normalized values. ​​**(C,E)**​​ Comparison of object exploration frequency between identical objects preoperatively versus POD3. ​​**(C)**​​ shows absolute counts, while ​​**(E)**​​ presents normalized values. ​​**(F)**​​ Movement trajectories demonstrating differential exploration patterns between novel (blue circles) and familiar (red circles) objects at POD3. ​​**(G,H)**​​ Novel object preference analysis: ​​**(G)**​​ exploration frequency and ​​**(H)**​​ duration comparing novel versus familiar objects at preoperative baseline and POD3. ​​**(I,J)**​​ Quantitative comparison of: ​​**(I)**​​ total exploration time and ​​**(J)**​​ frequency between novel and familiar objects at POD3. Data represent mean ± SD (n = 5 mice/group). Statistical significance was determined by two-tailed Student’s t-test (*P < 0.1).

**FIGURE 8:**
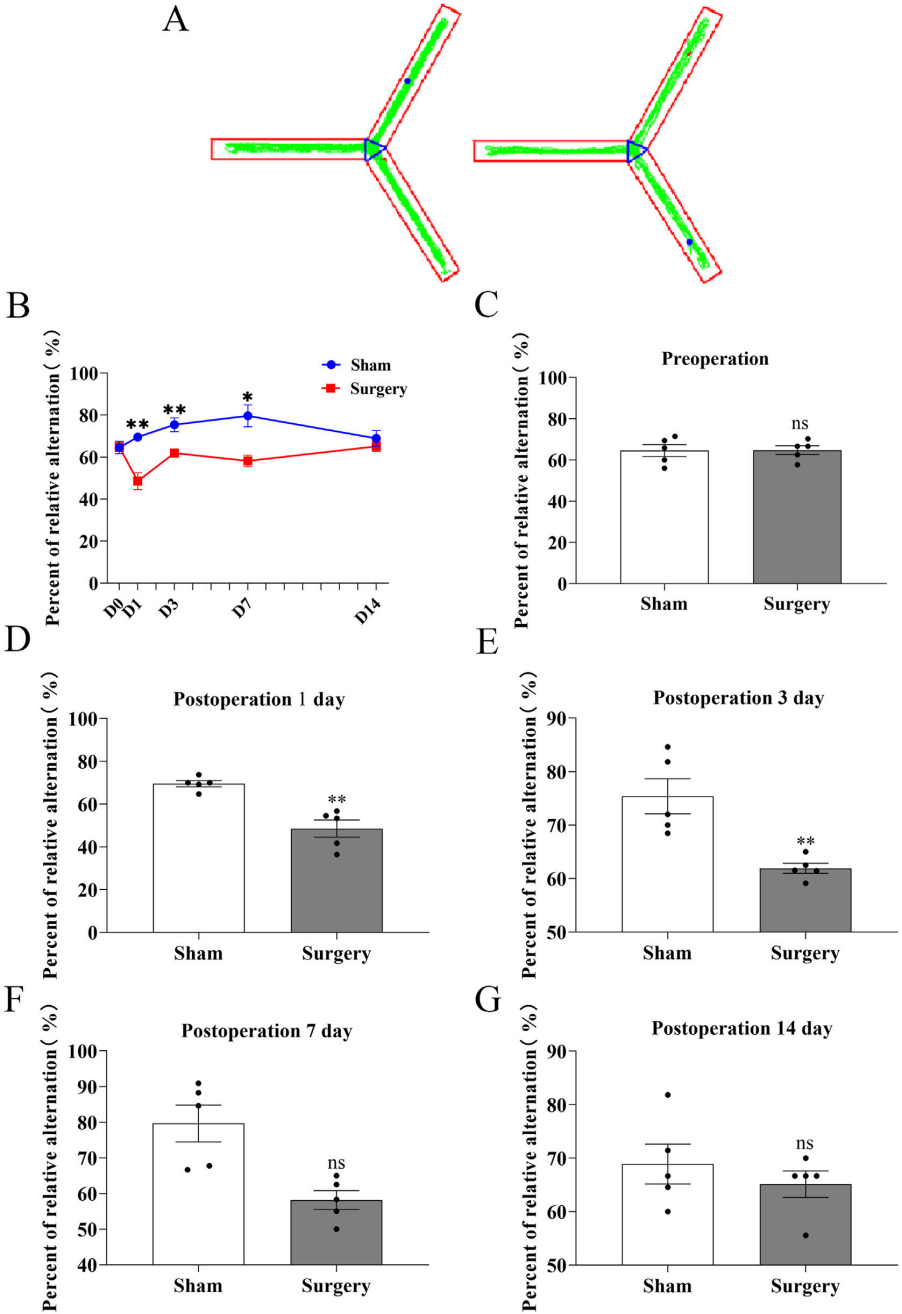
Assessment of working memory in POCD model mice using Y-maze spontaneous alternation test​​. ​​**(A)**​​ Representative movement trajectories of aged mice in the Y-maze apparatus, showing exploration patterns in both experimental groups. ​​**(B)**​​ Temporal progression of correct alternation rates (%) throughout the testing period, comparing baseline performance with postoperative time points (days 1, 3, 7, and 14). ​​**(C–G)**​​ Quantitative analysis of spontaneous alternation performance: ​​**(C)**​​ Preoperative baseline comparison; ​​**(D–G)**​​ Postoperative time points at day 1, 3, 7, and 14 respectively. Data represent mean ± SD (n = 5 mice/group). Statistical significance was determined by two-tailed Student’s t-test (​**​P < 0.01).

**FIGURE 9:**
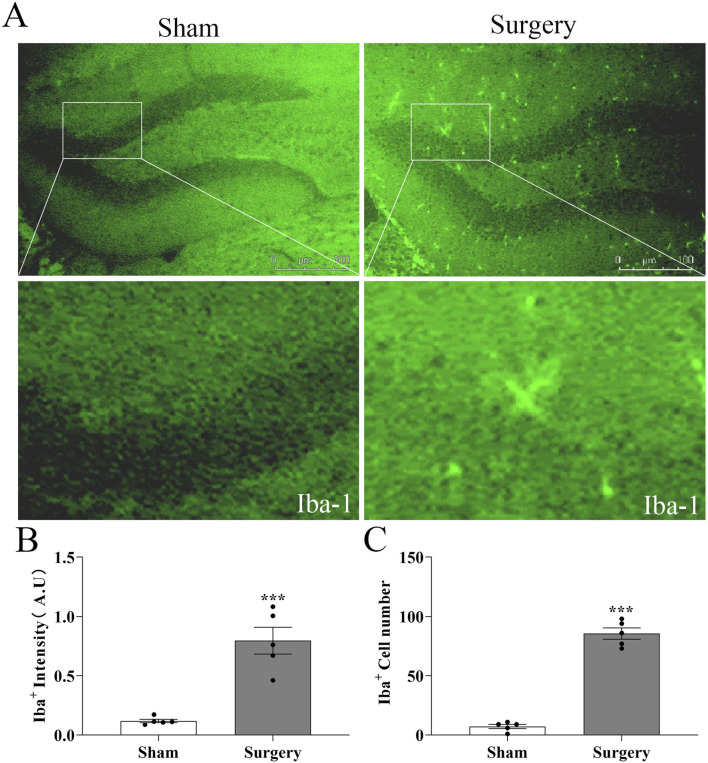
Microglial activation in hippocampal dentate gyrus of aged POCD mice​​. **​​(A)**​​ Representative immunofluorescence images showing Iba-1+ microglia in hippocampal sections from surgical and non-surgical groups at postoperative day 14 (POD14). Scale bar = 50 μm ​​**(B)**​​ Quantification of Iba-1 fluorescence intensity in the dentate gyrus region, demonstrating significant microglial activation in surgical versus control mice. ​​**(C)**​​ Statistical analysis of microglial cell counts in the dentate gyrus, confirming increased microglial proliferation in POCD mice. Data represent mean ± SD (n = 5 mice/group). Statistical significance was determined by two-tailed Student’s t-test (​**​*P < 0.001).

To establish a model of postoperative cognitive dysfunction (POCD), aged mice were subjected to tibial fracture surgery with intramedullary pin fixation. Mice in the sham group underwent identical procedures excluding the bone fracture and fixation. Cognitive function was assessed using the novel object recognition (NOR) test via Supermaze software, which tracks exploration time and trajectories. During the familiarization phase, no significant differences were observed between groups (Figures 7A–E). However, in the test phase, POCD model mice exhibited significantly impaired recognition memory and reduced exploratory preference for novel objects (Figures 7F–J), indicating cognitive deficits post-surgery. Cognitive impairment progression was further assessed via the Y-maze spontaneous alternation test (Figures 8A–G). The POCD group showed reduced alternation rates on postoperative days 1, 3, and 7, which normalized by day 14. Immunofluorescence staining of hippocampal tissue using the Iba-1 marker showed increased microglial activation in the dentate gyrus at day 14 (Figures 9A–C), confirming hippocampal inflammation in the POCD model.

### MCC950 improves cognitive impairment in SAA-Exacerbated POCD mice

**FIGURE 10:**
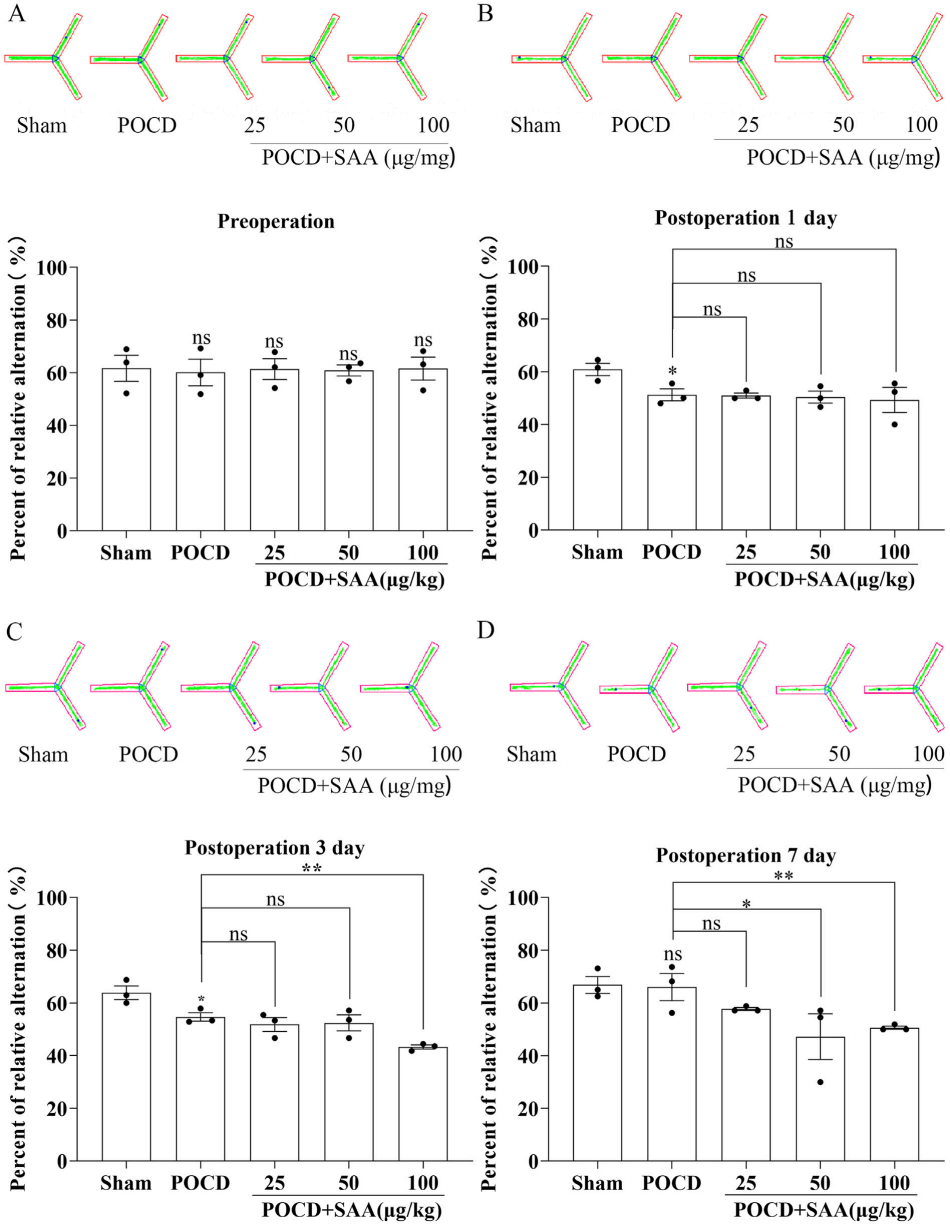
SAA impairs cognitive impairment in POCD model mice ​​. ​​**(A–D)**​​ Y-maze behavioral analysis showing: ​​**(A)**​​ Representative locomotion trajectories; ​​**(B–D)**​​ Quantification of spontaneous alternation rates (%) in: Sham-operated controls, POCD model mice, POCD mice treated with varying concentrations of SAA (0.1, 0.5, 1.0 μg/mL), POCD mice receiving MCC950 intervention. Data represent mean ± SD (n = 3 mice/group). Statistical significance was determined by one-way ANOVA followed by *post hoc* tests (*P < 0.1, ​** ​P < 0.01).

**FIGURE 11:**
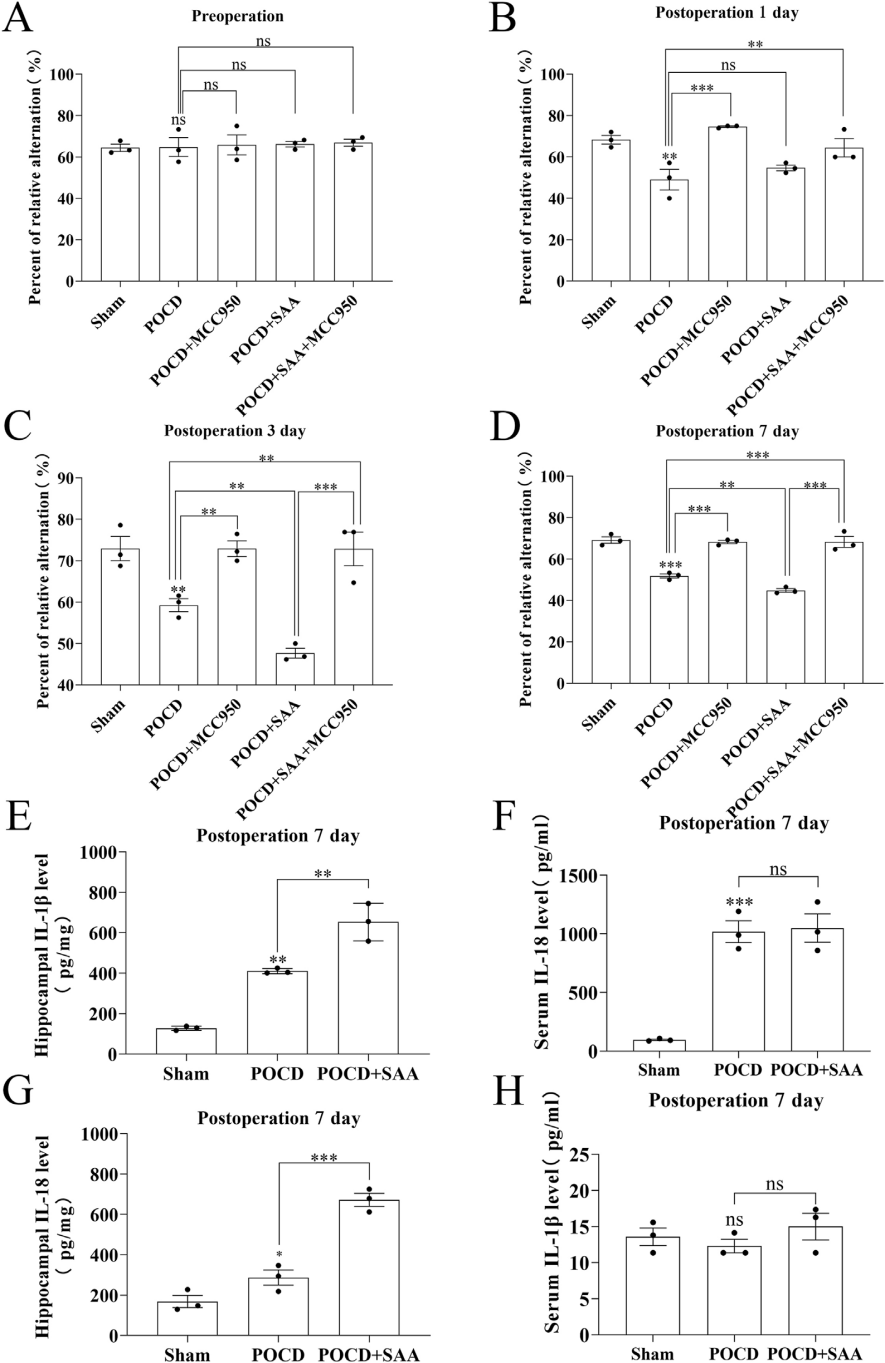
MCC950 treatment rescues cognitive deficits and neuroinflammation in POCD mice.​​ ​​**(A–D)**​​ Spontaneous alternation rates (%) in Y-maze test across groups: Sham-operated controls, POCD surgery model, POCD +100 μg/kg SAA, POCD + MCC950 (10 mg/kg), and POCD + SAA (100 μg/kg) + MCC950 (10 mg/kg). ​​**(E,F)**​​ IL-1β levels by ELISA in hippocampal tissue homogenates **(E)** and blood serum **(F)** 7 days post-POCD. ​​**(G, H)**​​ IL-18 levels in hippocampal tissue **(G)** and serum **(H)** at 7 days post-POCD. All data were expressed as Mean ± SD, one-way-ANOVA, *P < 0.05, **P < 0.01, ***P < 0.001, n = 3/group.

**FIGURE 12:**
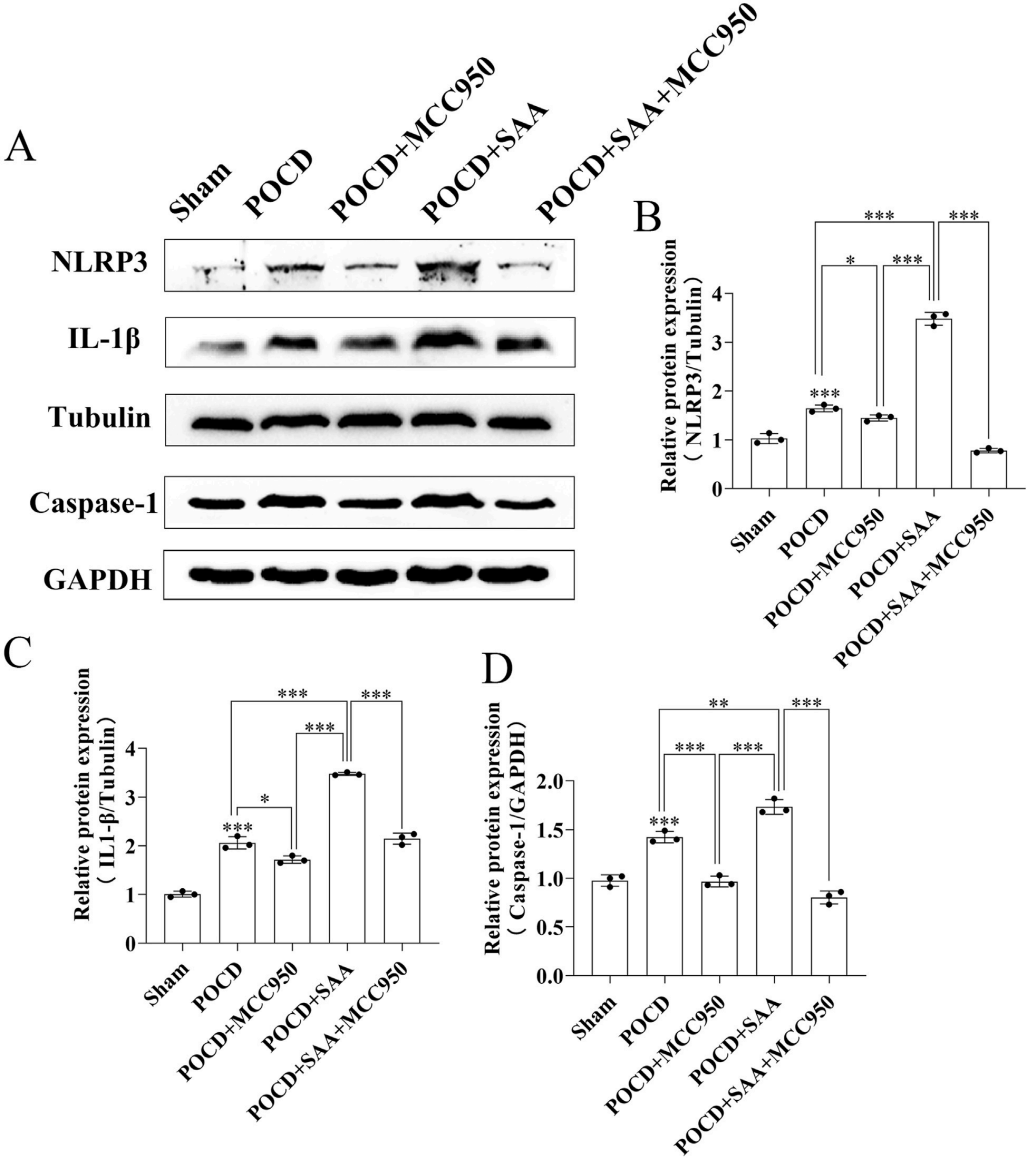
MCC950 modulates NLRP3 inflammasome pathway in POCD mice​​. ​​**(A)**​​ Western blot analysis of NLRP3 pathway proteins (NLRP3, IL-1β, Caspase-1) in hippocampal tissues from Sham, POCD model, POCD+100 μg/kg SAA, POCD+10 mg/kg MCC950, and POCD + SAA + MCC950 groups at 7 days post-modeling. ​​**(B–D)**​​ Quantitative analysis of protein expression normalized to Tubulin/GAPDH: ​​**(B)**​​ NLRP3, ​​**(C)**​​ IL-1β, ​​**(D)**​​ Caspase-1. All data are expressed as Mean ± SD, *P < 0.05, **P < 0.01, ***P < 0.001, one-way ANOVA, n = 3/group.

To evaluate the therapeutic potential of MCC950 in POCD, SAA was administered intraperitoneally at 25, 50, or 100 μg/kg every 3 days following surgery (Figure 10). The data showed that high concentration induced the cognitive impairment in surgical mice 3 days later (Figure 10). MCC950 (10 mg/kg/day) was administered postoperatively. Y-maze testing showed that MCC950 improved spontaneous alternation behavior in SAA-treated POCD mice (Figures 11A–D). Blood and hippocampal tissue collected on postoperative day 7 were analyzed for inflammatory markers. While SAA slightly increased serum IL-1β levels without reaching significance, serum IL-18 levels were significantly elevated (Figures 11F–H). In hippocampal tissues, both IL-1β and IL-18 were significantly upregulated following surgery and further increased with SAA administration (Figures 11E–G). Western blotting revealed significant upregulation of NLRP3, Caspase-1, and IL-1β in the hippocampus of POCD model mice (Figure 12). MCC950 treatment suppressed the expression of these inflammasome-related proteins, whereas SAA administration exacerbated their expression. These findings demonstrate that MCC950 effectively mitigates neuroinflammation and cognitive impairment in POCD mice, particularly in those subjected to additional SAA exposure.

## Discussion

SAA is synthesized and released into the bloodstream by activated macrophages and fibroblasts mainly in white adipose tissue and the liver and replaces apolipoprotein A1 (ApoA1), which binds rapidly to high-density lipoprotein (HDL) via the N-terminus (Sato et al., 2016). In healthy individuals, the concentration of SAA in the blood circulation is about 20–50 μg/mL, and after infection, it can be rapidly increased by about 1000 times within 4–6 h (Ye and Sun, 2015). Therefore, SAA can be used as a sensitive indicator to reflect the infection condition of the body and the effect of the treatment of inflammation. Non-glycosylated A-SAA has been identified as being locally expressed in normal tissue cells, tumor cells, chronic obstructive pulmonary disease, rheumatoid synovial tissues, atherosclerotic plaques, and neuroinflammatory lesions in the brains of Alzheimer’s disease patients. This suggests that SAA synthesized in extrahepatic tissue cells may function as an immune defense molecule, contributing to resistance against local inflammatory injury (Urieli-Shoval et al., 1998; Shridas and Tannock, 2019; Kumon et al., 1999). It is known that SAA concentrations may gradually increase with exacerbation of rheumatoid arthritis (Zhou et al., 2022). SAA has been shown to promote transendothelial LDL transcytosis via the NF-κB/caveolin-1/cavin-1 pathway during the pathophysiology of atherosclerosis (Peng et al., 2023). The SAA family of proteins has been highly conserved throughout evolution, with SAA1 and SAA2 being the acute-phase proteins (A-SAA) (Kindy et al., 2000). SAA4 encodes the constitutive SAA (C-SAA), whereas the SAA3 gene has been definitively demonstrated to be a pseudogene in humans but is expressed in mice, particularly in obese mice’s adipose tissue (Sack, 2018). In summary SAA plays a key role in the genesis and evolution of a wide range of diseases, however little is known about the role of SAA in the development of POCD.

SAA and Aβ are both amyloid proteins, and studies have reported elevated SAA concentrations in the cerebrospinal fluid of Alzheimer’s disease (AD) patients. Additionally, SAA has been shown to co-localize with Aβ deposits in the brain (Kindy et al., 1999). Guo et al. (2002) induced a systemic acute-phase response in SAA transgenic mice that enhanced Aβ deposition. There is no direct evidence of a direct functional link or interaction between Aβ and SAA, but both may play a role in their respective pathological processes, which can be the direction of our future research.

Studies have reported the design and synthesis of blood-brain barrier-penetrating peptides (BPPs) derived from the receptor-binding domain of apolipoprotein E (ApoE). These peptides were conjugated with phosphoramidomethyl morpholine oligomers (PMOs) to form BPP-PMOs, aiming to enhance the central nervous system (CNS) bioavailability of PMOs (Yeoh et al., 2024). However, this does not directly answer the question of whether Apo-SAA can enter the blood-brain barrier via intraperitoneal injection.

To directly Apo-SAA penetration, specialized experimental studies may be required, including intraperitoneal injections in animal models, followed by biochemical detection of the presence of Apo-SAA in brain tissue. We deduced that Apo-SAA can cross the blood-brain barrier or that its metabolites can produce such effects by the phenomenon of microglia activation in the hippocampal region of the mouse brain after intraperitoneal injection of Apo-SAA into mice. Recent studies have shown that SAA proteins can cross the intact blood-brain barrier into the brain and can impair BBB function (Erickson and Mahankali, 2024). However, the BBB transporter of SAA may also be inhibited by HDL, either through HDL binding or competition with other HDL lipoproteins (Erickson et al., 2017). Human serum amyloid A protein (apo-SAA) can be prepared by gel filtration of defatted acute-phase HDL in the presence of urea (Strachan et al., 1989). Apo-SAA was prepared by Shridas et al. (2018) by the method described above and isolated from plasma of patients 24 h after cardiac surgery or from lipopolysaccharide-injected mice by successive density gradient ultracentrifugation of human and mouse HDL. Notably, Shridas et al. also found that the ability of SAA to stimulate ROS generation and inflammatory vesicle activation was significantly attenuated after SAA incorporation into high-density lipoprotein (HDL). These results provide detailed insights into SAA-mediated IL-1β production and highlight the role of HDL in modulating the proinflammatory effects of SAA.

We applied the supernatant from SAA-stimulated BV-2 cells to primary neuronal cells *in vitro*, mimicking the interaction between microglia and neurons during the experimental process. This approach helps explain why SAA alone does not exhibit toxic effects on cell lines or primary neuronal cells, whereas the supernatant from SAA-stimulated BV-2 cells significantly increases apoptosis in primary hippocampal neurons. Tang et al. (2020) and his team also used the above mentioned experimental method of co-culturing the above mentioned conditions in order to demonstrate that melatonin attenuates thromboxane-induced inflammation in BV2 cells, protects HT22 cells from apoptosis.

Targeting the NLRP3 inflammasome has become an exploding area of research. The activation signals contain NLRP3 inflammasome activation, which induces spleen tyrosine kinase (SYK) and Bruton’s tyrosine kinase (BTK) to act on apoptosis-associated speck-like protein containing a CARD (ASC), positively regulating the protein hydrolysis of the caspase-1 and its downstream cytokine IL-1β maturation (Schwaid and Spencer, 2021). LPS has become a well-recognized activator of the NLRP3 pathway, and in our study, we were surprised to find that SAA exerted a similar activation of the NLRP3 pathway as LPS in the BV2 cell line at different temporal concentration gradients (Figures 1C–H, 2A–F) (Zhu et al., 2023). We further identified differentially expressed genes after SAA stimulation of BV-2 cells by transcriptomic analysis to reveal key targets of neuroinflammation induced by SAA stimulation. Screening of differentially expressed genes (DEGs) in normal control and SAA groups. mRNA transcriptome sequencing results showed that SAA promoted the upregulation of genes for a series of cytoskeleton-related proteins, such as inflammatory factors, actin filaments, etc., with significant differences in the expression of genes, such as IL-1β, Acta1, etc., which was in complete agreement with the results of immunoblotting experiments in our study.

POCD is a serious postoperative complication in elderly patients characterized by post-surgical confusion, memory loss, social impairment, disrupted sleep rhythms, fine motor difficulties, depression and even higher levels of cognitive impairment. The establishment of animal models is crucial to the study of the mechanisms and therapeutic approaches of POCD.

The POCD models usually require anesthesia and surgery on mice or rats. For anesthesia, inhalation anesthetics such as isoflurane, sevoflurane or desflurane, intravenous propofol, and intraperitoneal anesthetics such as isoproterenol or midazolam in combination with ketamine can be used to induce animal POCD models (Belrose and Noppens, 2019). Surgical procedures such as internal fixation of tibial fracture, cesarean section, cardiopulmonary diversion, hepatectomy/splenectomy, left nephrectomy, and appendectomy can induce animal models of POCD (Young et al., 2005; Xie et al., 2021; Zheng et al., 2019). Xu et al. (2023) investigated the effect of obesity on POCD by constructing an animal model of POCD by internal fixation of tibial fracture under isoflurane inhalation in 15-month-old mice, and concluded that obesity may exacerbate POCD by triggering oxidative stress-induced activation of the PARP1/NAD+/SIRT1 axis. Internal fixation of tibial fractures can achieve good consistency and stability, has no long-term adverse effects on important physiological functions in experimental animals, is easy to perform, and has a high success rate. Thus, we performed internal fixation of tibial fracture to establish a mouse model of postoperative cognitive dysfunction in old mice, and the postoperative behavioral indices of mice confirmed the success of the modelling (Figures 7, 8). Meanwhile, the relative fluorescence intensity of Iba-1 in the hippocampal region of the brains of the mice in the Surgery group was significantly enhanced, with altered morphology of microglia, which were significantly activated and mediated neuroinflammation (Figure 9). Ji et al. (2024) constructed the same mouse as our POCD model and assessed spatial reference memory by water maze (MWM) test. The results showed that the escape latency was significantly longer in the POCD group on day 3–5 and was longer than in the non-POCD group. Meanwhile, the time within the target quadrant and the time across the plateau were increased in POCD mice compared to non-POCD and non-surgical mice. HE staining was used to assess hippocampal injury, and the results showed that hippocampal neurons exhibited loose arrangement and disorganization in POCD mice. Feng (Feng et al., 2017) and members of his team, in order to examine the role of microglia in POCD, inhibited colony stimulating factor 1 receptor (CSF1R) to deplete microglia in adult mice based on internal fixation of tibial fractures. This study demonstrated that microglia depletion *per se* does not affect learning or memory, but that perioperative microglia depletion significantly protects mice from POCD, and therefore targeting microglia may be a viable strategy to attenuate the development of POCD.

The association between POCD and serum concentrations of IL-1β and S-100β persisted after adjustment for age and fetal bovine serum (FBS) (Yu et al., 2017). In this preliminary study, levels of inflammatory markers were significantly increased after treatment in adult patients, and this elevation was associated with the development of short-term cognitive dysfunction after this complex procedure. These results need to be replicated and confirmed in larger randomized controlled trials, but we are already intrigued by the possible in-depth link between SAA and POCD (Yu et al., 2017). Not surprisingly, in our primary hippocampal apoptosis index assay (Figures 5F,G, 6A–D) along with the results of the behavioral experiments (Figures 10–12), SAA significantly exacerbated the cognitive level of the POCD-modelled group of mice, whereas MCC950, an NLRP3 inhibitor, significantly suppressed the change. This suggests that SAA may exert its role in mediating neuroinflammation and thus affect cognitive function precisely through the NLRP3 pathway.

Of course, there are some shortcomings in this study, which did not directly reveal the effects of microglia on neurons by conditioning co-cultures of primary microglia directly with primary hippocampal neuronal cells. Haruwaka et al. (2024) demonstrate that microglia enhance neuronal activity after anesthesia by shielding inhibitory synapses, the Cx3cr1GFP/+(JAX: 005,582) mice for visualizing microglia and neuronal interactions following *in vivo* two-photon imaging. Experiments involved viral injections, chronic window implantation and synaptic reconstruction display with the aid of electron microscopy. This can also serve as an idea that we can build upon in future studies. The focus will be on the expression and role of mRNAs and proteins corresponding to different mammalian SAA under pathophysiological conditions, their potential activity in promoting disease progression, and their use as potential targets for therapy. Relevant clinical studies as well as preclinical models with SAA knockout, double knockout or even knock-in models should be considered to elucidate the adverse or beneficial effects of SAA on human pathology and other mammalian species. The fact that we used male mice for all the behavioral experiments in this experiment to rule out hormonal effects, and did not conduct relevant studies in female mice, is also an area we could investigate. In addition, we can also use microglia depletion agents to exclude the effects of microglia on mice. In addition, whether SAA has a similar effect to LPS in disrupting the blood-brain barrier deserves further investigation.

## Conclusion

In conclusion, our results suggest that SAA induces microglia inflammation *in vivo* and *in vitro* leading to an increase in neuronal apoptosis and consequently to a decrease in cognitive function in mice. The mechanism may be related to the regulation of the NLRP3 signaling pathway. The results suggest that NLRP3 inhibitors such as MCC950 may be a promising therapeutic candidate for neuroinflammation and may provide new inspiration for the development of therapeutic approaches for the treatment of various neurodegenerative diseases.

## Data Availability

The data presented in the study are deposited in the NCBI repository, with the following Object IDs: 52854064: https://www.ncbi.nlm.nih.gov/sra/52854064, 52854065: https://www.ncbi.nlm.nih.gov/sra/52854065, 52854066: https://www.ncbi.nlm.nih.gov/sra/52854066, 52854067: https://www.ncbi.nlm.nih.gov/sra/52854067, 52854068: https://www.ncbi.nlm.nih.gov/sra/52854068, 52854069: https://www.ncbi.nlm.nih.gov/sra/52854069, 52854064: https://www.ncbi.nlm.nih.gov/sra/52854064, 52854065: https://www.ncbi.nlm.nih.gov/sra/52854065, 52854066: https://www.ncbi.nlm.nih.gov/sra/52854066, 52854067: https://www.ncbi.nlm.nih.gov/sra/52854067, 52854068: https://www.ncbi.nlm.nih.gov/sra/52854068, 52854069: https://www.ncbi.nlm.nih.gov/sra/52854069.
